# Electron Beam Irradiation of Cellulosic Materials—Opportunities and Limitations

**DOI:** 10.3390/ma6051584

**Published:** 2013-04-29

**Authors:** Ute Henniges, Merima Hasani, Antje Potthast, Gunnar Westman, Thomas Rosenau

**Affiliations:** 1 Department of Chemistry/Division of Chemistry of Renewables, University of Natural Resources and Life Sciences, Vienna A-1190, Austria; E-Mails: ute.henniges@boku.ac.at (U.H.); antje.potthast@boku.ac.at (A.P.); 2 Department of Chemical and Biological Engineering/Organic Chemistry, Chalmers University of Technology, Gothenburg SE-412 96, Sweden; E-Mails: merima.hasani@chalmers.se (M.H.); westman@chalmers.se (G.W.)

**Keywords:** carbonyl groups, carboxyl groups, crystalline cellulose, degradation, fibrillated cellulose, molar mass

## Abstract

The irradiation of pulp is of interest from different perspectives. Mainly it is required when a modification of cellulose is needed. Irradiation could bring many advantages, such as chemical savings and, therefore, cost savings and a reduction in environmental pollutants. In this account, pulp and dissociated celluloses were analyzed before and after irradiation by electron beaming. The focus of the analysis was the oxidation of hydroxyl groups to carbonyl and carboxyl groups in pulp and the degradation of cellulose causing a decrease in molar mass. For that purpose, the samples were labeled with a selective fluorescence marker and analyzed by gel permeation chromatography (GPC) coupled with multi-angle laser light scattering (MALLS), refractive index (RI), and fluorescence detectors. Degradation of the analyzed substrates was the predominant result of the irradiation; however, in the microcrystalline samples, oxidized cellulose functionalities were introduced along the cellulose chain, making this substrate suitable for further chemical modification.

## 1. Introduction

Its natural abundance highlights cellulose as one of the most promising renewable raw materials for the production of functional materials in a bio-based economy beyond its conventional mass commodity uses (e.g., for the development of special papers, composites, transparent films, and packaging products). Cellulosic materials, such as microcrystalline (MCC), nanofibrillated celluloses (NFC), and cellulose whiskers, seem to have the potential for even more intriguing applications. However, some processing techniques, such as fiber modification, are required to achieve new properties for innovative materials and their applications. Among these techniques, irradiation may be an interesting approach. Although relatively energy-intensive, treatment times are short in general, and no specific sample preparation or chemicals are needed. This also means that related purification procedures are obsolete, and no residual chemicals are left in the sample—provided that no additives were used during the irradiation treatment. 

Ionizing radiation can either originate from a radioactive source or from highly accelerated electrons; both are able to replace a chemical treatment for the modification of polymers. Computer simulations of electron beam-irradiated powdered cellulose samples suggest that three of several free radical species are present simultaneously [1]. Another study concluded from findings of the life time of radicals that electron beam irradiation increases the reactivity of the cellulose pulp towards reagents [2].

Usually, electron beams are deployed in research, technology, and medical therapy to produce images on television screens. Also, they are used for electron microscopes, and hence for the analysis of materials. For commercial use, the most important characteristics of an accelerator are its electron energy and average beam power. Therefore, industrial electron accelerators are usually classified according to their energy range, which are divided into low- (80–300 keV), medium- (300 keV–5 MeV), and high-energy ranges (above 5 MeV). Today, irradiation facilities can offer a broad range of treatment options, often custom-made.

The effects of electron beam irradiation on cellulose have been evaluated in several studies [3,4,5]. All agree that irradiation causes cellulose depolymerization. One of the first studies of cellulose interaction with high-energy radiation was reported in 1952. It identified chain scission and reduced crystallinity as the most prominent effects. Saeman *et al.* noted a considerable introduction of oxidized groups upon irradiation of cellulose while making an effort to quantify the amount of introduced carboxylic acid groups [6]. Some authors also observed an increase in carbonyl group content [7,8]. Further investigations of oxidizing effects were rather neglected as the focus was traditionally on degradation associated with irradiation, rather than on detailed mechanisms or usage for chemical modification. Irradiation-mediated cellulose chain cleavage has been of special interest to industry as a promising method to enhance the conversion of biomass to monomers and fuels [9], and to increase the reactivity of dissolving pulps in derivatization procedures in general, and in viscose production in particular [10,11]. Furthermore, efforts on developing new functionalization techniques for textile fibers have increasingly included various radiation mediated treatments [1,12,13,14].

In addition, it is difficult to selectively oxidize the cellulose backbone. Mostly, different oxidized functionalities (carbonyls—keto and aldehyde groups also as hydrates, hemiketals and hemiacetals—and carboxyls) tend to originate in addition to newly generated reducing end groups (REGs) formed upon chain scission. Consequently, for modifying cellulose, only mild irradiation can be applied, otherwise the negative impact caused by chain scission will render the irradiated material mechanically too weak [15]. Another aspect of electron beam irradiation refers to the decrease of the amount of I-alpha cellulose [4]. The amount of the I-alpha allomorph continuously decreases with increasing dosages of electron beam irradiation. Moreover, the conversion of the I-alpha form to the I-beta form is less at the higher dosage (10 MeV). However, the degree of crystallinity was found to be unaffected by irradiation [16]. It is evident that several aspects of the interaction between cellulose and electron beam irradiation are still far from being sufficiently understood.

In this study, different pre-treatments were applied to cellulosic pulp to see if they are able to favor oxidation upon electron beaming while conventional irradiation is dominated by chain scission. Further, the impact of electron beam irradiation was studied on “dissociated” cellulosic materials with the aim of better understanding the viability of using irradiation to modify crystallites or nanofibers. The term “dissociated” cellulosic material is introduced in this study to refer to microfibrillated, nanofibrillated, microcrystalline, and nanocrystalline cellulosic materials.

## 2. Results and Discussion

### 2.1. Modification of Irradiation Treatments

**Figure 1:**
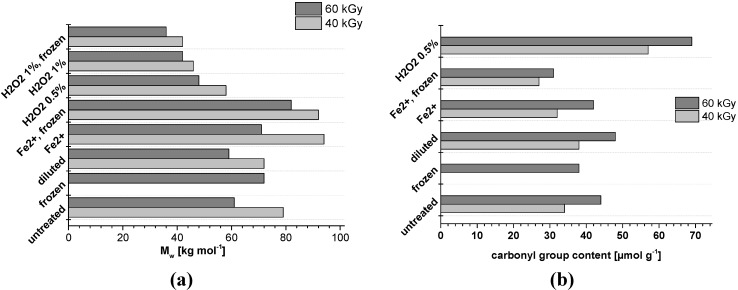
Modification of irradiation treatments and their impact on pulp with regard to weight average molar mass (**a**) and to carbonyl group content (**b**).

One interesting question is whether the irradiation impact can be tuned not only by adjusting the irradiation dosage and energy source, but also by the addition of certain additives or preparation methods before irradiation. The efficiency of degradation increases considerably with temperature and depends on the structure of the polysaccharide and the nature of its substituents [3]. When it comes to treatments in aqueous suspensions, the formation and the consecutive reactions of the strongly oxidizing hydroxyl radicals are crucial for the outcome of the oxidation processes. For example, radiation degradation of the fibers irradiated under humid conditions was less than that irradiated under a vacuum [17]. The presence of oxidizing or easily oxidized species is expected to enhance the formation of hydroxyl radicals, whereas, at the same time, chain degrading side reactions have to be suppressed. In this context, several treatments have been applied to a pulp sample before irradiating them (Figure 1). 

Compared to the untreated sample, the dilution of the pulp suspension did not change the influence of irradiation to any large extent with regard to molar mass stability or carbonyl group development. Cooling improved the cellulose stability in general and in the presence of Fe^2+^ ions; for samples with H_2_O_2_, there was no such influence of cooling on the cellulose molecule. While the addition of Fe^2+^ ions improved the molar mass stability, there was no change with regard to the carbonyl group development. The addition of hydrogen peroxide diminished the molar mass stability and increased cellulose oxidation in all cases.

### 2.2. Irradiation of Crystalline Cellulose Structures

In general, more cellulose degradation is expected in the amorphous regions of the cellulose molecule than in the well-aligned molecules in crystalline regions. However, after electron beam irradiation with a dosage of 100 kGy, the molar mass of micro-crystalline cellulose (MCC) was reduced drastically from 82,000 to about 5400 g mol^−1^. No significant effect was observed after a dosage of 10 kGy in the same study. The relative crystallinity of the MCC was reduced from 87% to 45% with a dosage of 1000 kGy. The available surface area, an indication of how well cellulose will react with chemical agents, was increased from 274 m^2^ g^−1^ for the control sample (0 kGy) to 318 m^2^ g^−1^ at a dosage 1000 kGy [18].

**Figure 2:**
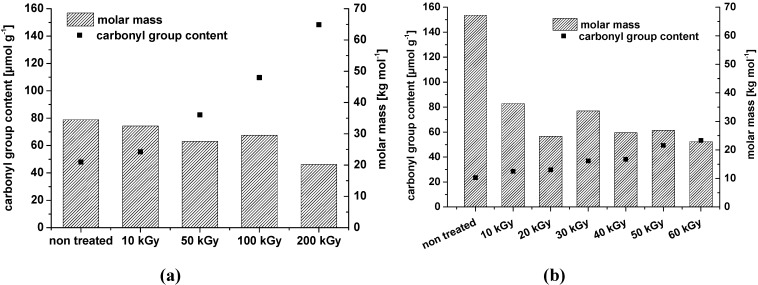
Weight average molar mass and carbonyl group content of microcrystalline cellulose (**a**) and nanocrystalline cellulose ((**b**) dry irradiation) after treatment with different dosage of two distinct electron beam irradiation sources (10 × 10^6^ *vs.* 300 × 10^3^ eV).

Comparable results were found in our own experiments where it was observed that irradiation can even oxidize microcrystalline and nanocrystalline cellulose. Also, there is a clear decrease in the weight average molar mass (Figure 2). 

Carbonyl-selective labeling with the marker carbazole-9-carboxylic acid [2-(2-aminooxyethoxy)ethoxy]amide (CCOA), when performed heterogeneously, reports the oxidative state of the accessible, mainly amorphous areas of the cellulose. By contrast, labeling in a homogeneous medium (*i.e.*, in dissolved state) covers the bulk material, *i.e.*, amorphous and crystalline regions as well. Thus, the two variants of the labeling procedure allow for differentiating between the amorphous and crystalline regions with regard to their oxidative modification. In cellulosic pulps, homogeneous labeling usually does not provide additional information, since no significant differences between amorphous and crystalline areas are present. This is why heterogeneous labeling, which is less time-consuming, is commonly preferred over the more labor-intensive homogeneous labeling [19]. However, for the highly ordered nanocrystalline cellulose (NCC) after irradiation, a homogeneous labeling did seem necessary in order to assess changes in the crystalline areas.

**Figure 3:**
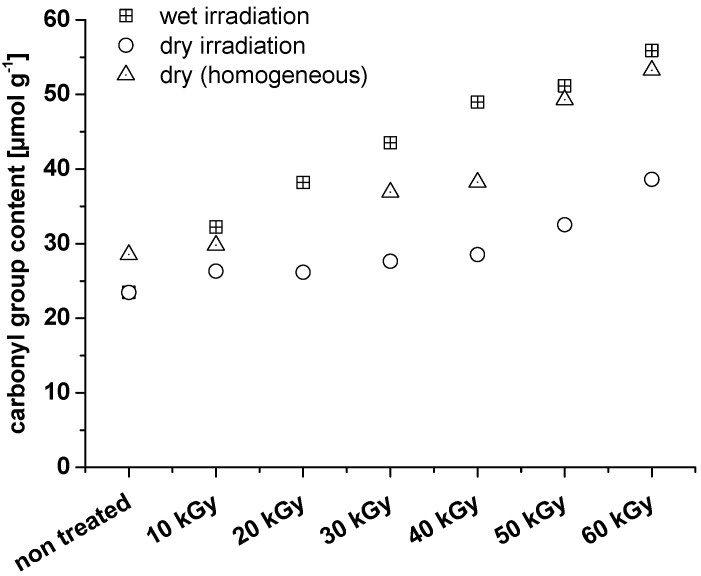
Comparison of nanocrystalline celluloses that have been irradiated as wet or dry material. The dry material has additionally been analyzed after homogeneous labeling.

It is obvious from Figure 3 that irradiation is able to inflict oxidation in the crystalline regions. The carbonyl values determined by homogeneous labeling are clearly higher than the values determined by heterogeneous labeling. In both cases, carbonyls in amorphous regions are reported, but homogeneous labeling also reports carbonyls in crystalline areas. Evidently, the latter are responsible for the observed increase in the overall carbonyl values. From the literature, results from wide-angle X-ray diffraction (WAXD) and Fourier transform infrared spectroscopy (FTIR) show that the crystalline structures of bamboo cellulose were not destroyed with an absorbed dosage of irradiation ranging from 0 to 60 kGy [20]. It must be concluded that oxidation by electron beaming under the pertinent conditions also affected the crystalline areas without significantly changing the crystalline structure. The amount of carbonyl groups introduced is too small to change the hydrogen bond network in a way that regular alignment of the cellulose chains (=crystallinity) is significantly disturbed. Nevertheless, in contrast to cellulosic hydroxyls, which can act as both hydrogen bond donors and acceptors, a carbonyl group (oxidized hydroxyl group) is only able to act as a hydrogen bond acceptor. Thus, there is a definite influence on the hydrogen bond network; it just remains too small to affect the overall crystallinity. With regard to an increase of carbonyl groups, the question arises whether these groups actually originate from oxidation along the cellulose backbone, or if the increase in carbonyl groups mostly reflects the freshly generated REGs that form upon chain scission. Therefore, based on the number average molar mass, the amount of REGs was calculated and compared to the analytically determined total amount of the carbonyl groups.

**Figure 4:**
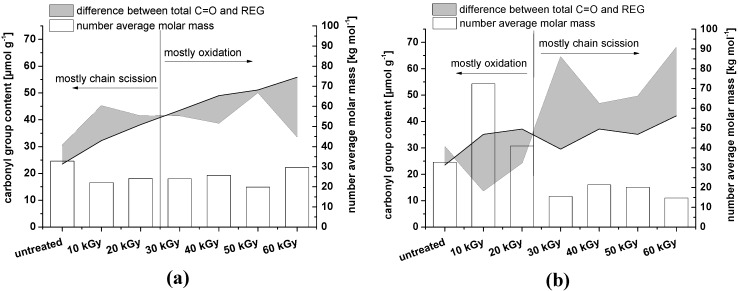
Content of REG compared to the total amount of carbonyl groups (determined by fluorescence labeling). The shaded area corresponds to the difference between total C=O and REG, representing oxidation on the cellulose backbone (when the shaded area expands below the black line). As the calculation of REG is based on the number average molar mass (*M*_n_), the data are shown as well (empty bars). (**a**) NCC wet; (**b**) NCC dry. The untreated material is identical in the two graphs.

It is clear that when irradiating dry NCC samples, oxidation along the cellulose chains can be achieved with dosages below 30 kGy, whereas irradiation of wet samples requires higher irradiation dosages for a significant oxidizing effect (Figure 4). It should be kept in mind, however, that in the case of dry NCC, the *M*_n_ value is probably overestimated due to inaccuracies in multi-angle laser light scattering (MALLS) processing and difficulties in NCC dissolution. The *M*_n_ of the samples after 10 and 20 kGy irradiation is expected to be approximately 20,000 g mol^−1^, as for the other samples. An overestimation of the *M*_n_ will turn into an underestimation of the REG. Therefore, in the case of NCC that was irradiated dry, it can even be assumed that no oxidation along the cellulose chain will occur at all. 

**Figure 5:**
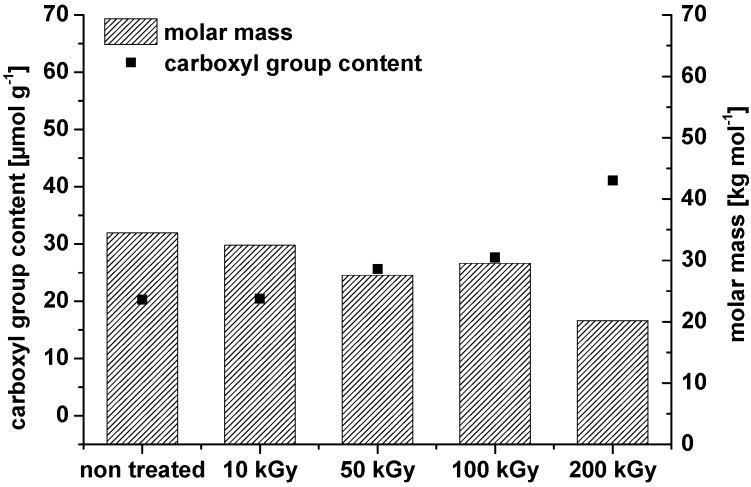
Weight average molar mass and carboxyl group content of microcrystalline cellulose after treatment with different dosage of electron beam irradiation.

Next to an increase in carbonyl group content, further oxidation to carboxyl groups is observed and follows the expected trend for MCC (Figure 5). The increase of carboxyl groups is, however, slower than the one observed for carbonyl groups: while the carbonyl group content increases about 3-fold, the carboxyl group content only increases about 2-fold.

**Figure 6:**
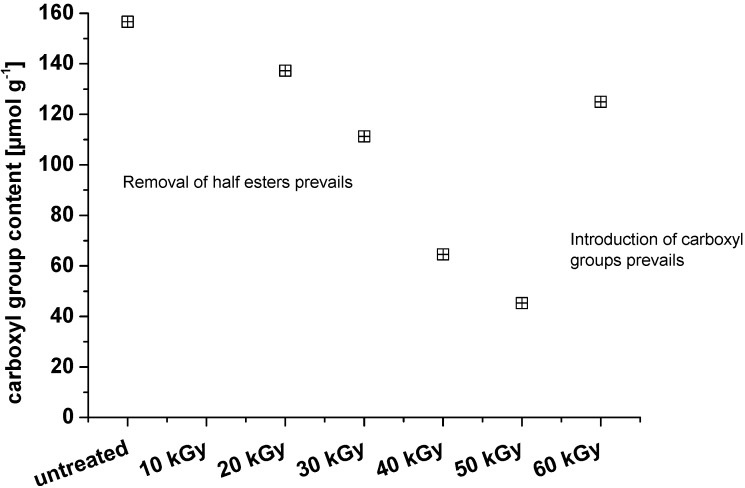
Changes in carboxyl groups of nanocrystalline cellulose upon increasing irradiation dosages.

The results of carboxyl group labeling for the NCC samples are severely influenced by the presence of sulfate half esters on the surface of these nanoparticles. Presumably, these unstable half esters are removed to various extents during the irradiation, while the remaining ones are likely to interfere with the labeling procedure, giving rise to similar responses as introducing carboxylic acid groups. Thus, the results of the labeling analysis (Figure 6) most likely reflect the sum of two parallel processes: removal of sulfate half esters during irradiation and increase in carboxylic acid groups. 

### 2.3. Irradiation of Fibrillated Celluloses

**Figure 7:**
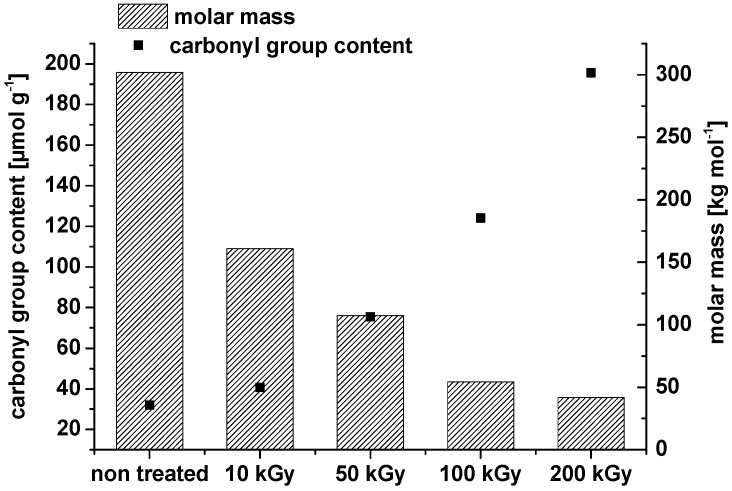
Freeze-dried nanofibrillated cellulose after electron beam irradiation of different dosages.

Irradiation of nanofibrillated samples caused an increase in oxidized functionalities and a decrease in molar mass. Nanofibrillated celluloses consist of crystalline and amorphous areas as do native cellulose samples. Therefore, results from this type of sample are comparable to native cellulose (Figure 7).

**Figure 8:**
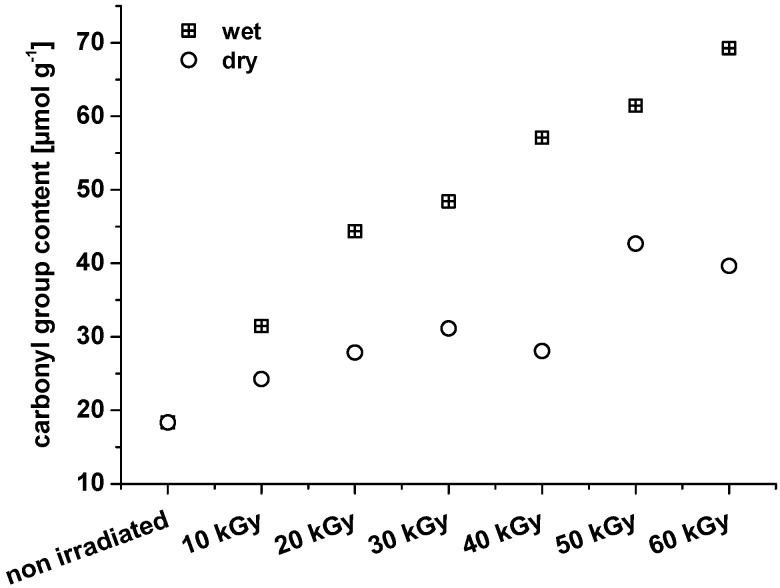
Development of the total carbonyl group content in MFC upon electron beam irradiation in wet and in dry conditions.

As in the case of the crystalline samples, the fibrillated samples were also subject to electron beam irradiation in both wet and dry conditions (Figure 8). In this sample set, more oxidation occurred in the wet irradiated samples than in the dry irradiated samples. According to the literature [17], humidity should favor the recombination of radicals formed upon irradiation. This is true for the absence of co-reactants that can be oxidized by irradiation-triggered radicals: if there are no other co-reactants, water provides a medium for the radicals to encounter each other and to recombine. In the presence of co-reactants, however (and this also means also the presence of cellulose), water first of all constitutes the medium for radical motion, enabling them to approach to and react with co-reactants. Most importantly, the lifespan of oxygen-derived radicals in water, such as hydroxyl, hydroperoxyl, and peroxyl anion radicals, is up to four times greater in magnitude than in the dry or gaseous medium [21,22]. This extension is the effect of solvation and (partially) charge stabilization, which is active for both ions and radicals. This explains the increased oxidation effects in wet cellulosic materials compared to dry counterparts. Humidity can also facilitate the oxidation compared to chain degradation, as indicated for the NCC samples above. 

Wet irradiation favors the oxidation of the microfibrillated cellulose (MFC); not only is the total number of oxidized functionalities higher, but there is also a positive deviation when it comes to the difference between total carbonyl groups and REG, pointing to the opportunity of actually introducing carbonyl functionalities along the cellulose molecule “without chemicals”. In this case, the reactivity of cellulose towards linking additional compounds to it increased: the introduced functionalities may be used as reactive anchor groups in cellulose modification.

**Figure 9:**
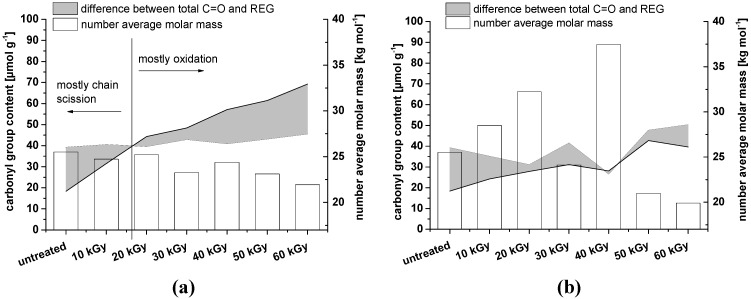
Content of REG compared to the total amount of carbonyl groups (determined by fluorescence labeling). The shaded area corresponds to the difference between total C=O and REG, representing oxidation on the cellulose backbone (when the shaded area expands below the black line). As the calculation of REG is based on the number average molar mass (*M*_n_), the data are shown as well (empty bars). (**a**) MFC wet; (**b**) MFC dry. The untreated material is identical in the two graphs.

As already observed for NCC, irradiation under dry conditions predominantly favors scission of the cellulose chain (Figure 9). In the case of MFC, this effect is even more pronounced as a consequence of a substantial amorphous content. 

**Figure 10:**
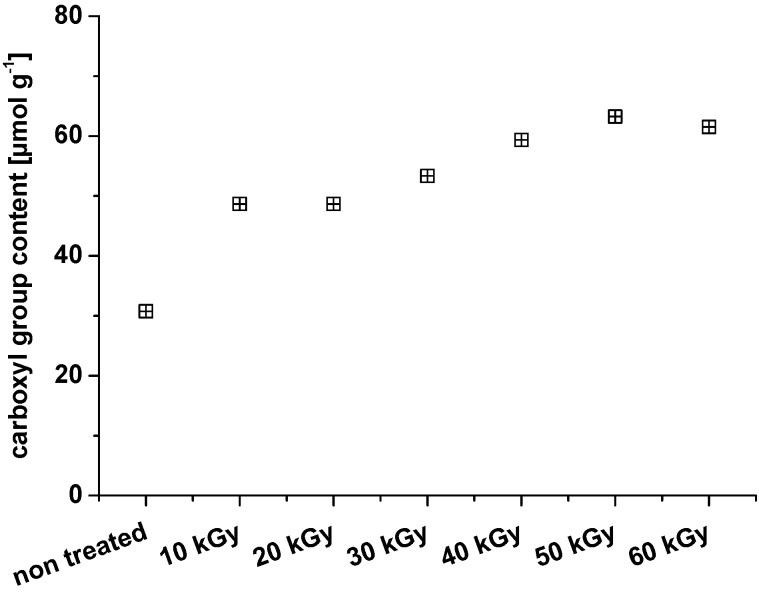
Development of the carboxyl group content in MFC after irradiation with increasing dosages.

Next to a clear increase in the carbonyl group content, also the carboxyl group content increased (Figure 10). This fosters additional opportunities to link compounds to the MFCs. 

### 2.4. Impact of Irradiation Treatment on Solubility

According to the yet unpublished results by the authors, it was pointed out that dissociated celluloses are difficult to dissolve, and ways to tackle this problem were proposed [23]. Irradiation may change the solubility properties; at least enzymatically treated, electron beam-irradiated pulps from different sources became more readily soluble in an aqueous sodium hydroxide (NaOH) solution than those treated by gamma irradiation or by alkaline pre-treatment [24].

Rates of dissolution of irradiated and non-irradiated MFC samples in *N*,*N*-dimethylacetamide/ lithium chloride (DMAc/LiCl) indicate a certain activation of the samples by low-dosage irradiation (Table 1). This activation is most likely achieved in terms of increased accessibility for the solvent and weakened hydrogen bond networks that translate into better solubility. At higher irradiation dosages this effect is suppressed by cross-linking (intra- and intermolecular hemiacetals and hemiketals). These crosslinks are sufficiently stable to impede dissolution, but obviously too labile to survive the acidic labeling conditions [25]. 

Interestingly, low irradiation dosages proved to be a more efficient tool in assisting dissolution than the ethyl isocyanate (EIC) method [26], which the authors previously reported to aid the dissolution of softwood kraft pulp. This indicates the ability of the irradiation to activate even crystalline areas which may not be accessible to the EIC reagent during heterogeneous derivatization conditions. The mechanism of this irradiation activation must again be assumed to be the weakening of the hydrogen bond network, in which hydroxyl groups (H-donating and H-accepting) are converted into carbonyls (only H-accepting). 

It seems that the solubility of NCC is improved by low irradiation dosages that are efficient enough to introduce imperfections into the crystals (Table 2). As already observed for the MFC sample, the effect on dissolution is the opposite for higher dosages.

**Table 1:** Impact of irradiation on the solubility of MFC produced by mechanical fibrillation of northern bleached softwood kraft (NBSK) pulp (according to standard dissolution procedures as described in Section 3.4 and ethyl isocyanate (EIC) supported dissolution).

MFC-material	After 24 h in 9% DMAc/LiCl	After 72 h in 9% DMAc/LiCl	EIC-supported dissolution ^2^ after 48 h	EIC-supported dissolution after 672 h
untreated	−	+	−	−
10 kGy	−	+	−	−
20 kGy	−	+	+	+
30 kGy	−	+/− ^1^	+	+
40 kGy	−	+/− ^1^	+	+
50 kGy	−	+/− ^1^	+	+
60 kGy	−	−	+	+

note: ^1^ Turbid viscous “solution”/homogeneous suspension—no solid material left; ^2^ The results did not change upon doubling the amount of EIC and shaking the samples at 40 °C overnight; “+”: for dissolved samples; “−”: for not dissolved: “+/−”: for partially dissolved.

**Table 2:** Dissolution of NCC (prepared from Whatman cotton filter aid) according to standard solution procedure ^2^.

NCC material	After 48 h	After 336 h	After 336 h + freezing and thawing
Untreated	−	−	+
10 kGy	+	+	+
20 kGy	+/− ^1^	+	+
30 kGy	−	+/− ^1^	+
40 kGy	−	+/− ^1^	+
50 kGy	−	−	+
60 kGy	−	−	−

Note: ^1^ Turbid viscous “solution”/homogeneous suspension—no solid material left; ^2^ The amount of added DMAc/LiCl per weight of NCC was doubled compared to the standard procedure (1 ml DMAc/LiCl (9%) per 10 mg dry sample); “+”: dissolved; “−”: not dissolved; “+/−”: partially dissolved.

## 3. Experimental Section

### 3.1. Samples

Dissociated celluloses are not readily available, therefore, it was decided to thoroughly characterize their starting materials. To address the question of irradiation modification in general, pre-hydrolysis kraft dissolving pulp from eucalyptus was used.

For preliminary experiments about the impact of electron beams on dissociated celluloses, commercially available microcrystalline cellulose (MCC) and nanofibrillated cellulose (NFC) samples were used. MCC was obtained from JRS Pharma. According to the producer, the average particle size by laser diffraction is 190 µm. The product is mainly used as an insoluble tablet binder for pharmaceutical products. NFC from JRS used in this experiment was freeze-dried to reduce interaction between the nanofibrils.

More specific experiments were performed on NCC obtained by sulfuric acid hydrolysis (from the Whatman cotton filter aid No. 541) as described elsewhere [27]. The average dimensions of the nanocrystals are (2–5) nm × (100–200) nm. MFC was obtained from the Norwegian Paper Research Institute (PFI) prepared by extensive refining of NBSK pulp, followed by three high-pressure homogenization passes (BlueTop MT20.70H, 1–89.200 from SPX) at 1% concentration and 1000 bar.

### 3.2. Irradiation Treatments

The irradiation treatments were performed at two different facilities, chosen according to availability. Different irradiation energies and dosages were applied. 

First, electron beam irradiation was conducted at NHV Corporation in Kyoto, Japan, by a Curetron^®^ EBC 300-60 radiation apparatus. The powdered and granulated samples were spread inside PET plastic bags during the irradiation. The samples went across the beam flow twice (upside down) at 300 keV, and were irradiated at dosages of 10, 50, 100, and 200 kGy.

The second electron beam irradiation facility is based at Kremsmünster, Austria. This facility uses a Rhodotron IBA TT-100 accelerator at 10 MeV. The precise dosage was determined to deviate within ±0.4% from the nominal dosage. Dosages between 10 and 60 kGy were used as indicated in the individual experiments.

### 3.3. Modified Irradiation Treatments

For investigation of modified irradiation treatments, 15% aqueous suspensions of eucalyptus dissolving pulp were irradiated at ambient temperature or as frozen samples at dosages of 40, 60, and 90 kGy, containing either of the following additives: 0.5% H_2_O_2_, 1% H_2_O_2_, or 0.004% Fe_2_SO_4_. To study the effect of dilution, one set of samples was irradiated as 7.5% aqueous suspensions. Freezing of the samples was performed at the irradiation facilities so that the irradiation process started on frozen samples that gradually thawed upon heating during irradiation.

### 3.4. General Sample Work-up

For fluorescence labeling of all cellulosic materials, the amount of the sample corresponding to 10 mg of dry material was weighed into separate 4-mL vials. Some of these samples consisted of a fine powder; thus, they could not be worked up as described in the literature. In this case, after settling of the sample powder, the water or the chemicals were pipetted out of the vials. Afterwards, they were rinsed with ethanol by two repeated centrifugations. The samples irradiated in the dry state were also suspended in water and subjected to a similar procedure in order to enhance their reactivity. Subsequently, carbonyl groups were “CCOA” labeled and carboxyl groups “FDAM” labeled, as described in the paragraph below and in the literature [19,28]. In brief, the two markers selectively react with either the carbonyl or carboxyl groups. The quantification is performed by simultaneous fluorescence and RI detection. The normalized signals are used to build a calibration line based on known standard pulps.

In order to study the dissolution of irradiated dissociated cellulosic materials without further fluorescence labeling, sample amounts corresponding to 20 mg dry material were dewatered, rinsed with ethanol, and the solvent was exchanged to DMAc using repeated centrifugations. The subsequent dissolution treatment was performed according to the common procedures and included an overnight activation in DMAc followed by a wash with DMAc and a final dispersion in 9% (w/v) DMAc/LiCl.

The progress of dissolution was followed qualitatively by visual observations.

### 3.5. Analytical Set-up

GPC measurements used the following components: online degasser, Dionex DG-2410; Kontron 420 pump, pulse damper; auto sampler, HP 1100; column oven, Gynkotek STH 585; MALLS detector, Wyatt Dawn DSP with argon ion laser (λ_0_ = 488 nm); fluorescence detectors, Shimadzu RF 535 (λ_ex_: 280 nm, λ_em_: 312 nm) and TSP FL 2000 by Spectra Physics (λ_ex_: 290 nm, λ_em_: 340 nm); RI detector, Shodex RI-71. Data evaluation was performed with standard Astra, GRAMS/32, and Chromeleon software.

The following parameters were used for GPC measurements: flow: 1.00 mL min^−1^; columns: four PL gel mixedA LS, 20 µm, 7.5 × 300 mm; injection volume: 100 µL; run time: 45 min; DMAc/LiCl (0.9% w/v), filtered through a 0.02 µm filter, was used as mobile phase.

The amount of dissolved material was determined from the RI signal using a *d*n/*d*c of 0.136 mL g^−1^ and a detector constant of 5.3200 e^−5^ V^−1^; both values were determined in the lab of the University of Natural Resources and Life Sciences, Vienna, Austria.

## 4. Conclusions 

The irradiation of dissociated celluloses both offers opportunities and imposes limitations. First, some logistic operations are involved in the process of treating the samples. This requires careful planning and implies economic considerations with regard to time and expense. However, irradiation of food and medical equipment is standard nowadays and may serve as an example for cellulosic materials.

From the first part of this study, we conclude that tuning of the irradiation process, independent of the dosage, is possible with an increase of oxidation by adding iron(II) ions. Here, we observe the same amount of oxidized cellulose functionalities at a better preservation of molar mass (less cellulose chain degradation). The addition of hydrogen peroxide on the other side accelerates the degradation process caused by irradiation in any case.

Another conclusion is that wet irradiated samples are more affected than those that are dry irradiated. When looking at the theoretical amount of REG that can be calculated from the number average molar mass (*M*_n_), it becomes obvious that there is not much oxidation of the cellulose backbone occurring, but rather an increase of the oxidized functionalities caused by newly formed REG.

Irradiation has a considerable impact on dissociated celluloses, leading to an increase of oxidized groups also in the crystalline areas, and a corresponding weakening of the hydrogen bond network. This is probably one of the reasons why irradiation can facilitate the dissolution of the dissociated celluloses in DMAc/LiCl, which is generally difficult to achieve for this type of samples. 

Another aspect is the “chemical activation” of celluloses by irradiation (*i.e.*, providing carbonyl and carboxyl functionalities to add specific molecules and, thus, specific properties to the polymer). For that purpose, micro- or nano-fibrillated celluloses after wet irradiation seem to be much more suitable than nanocrystalline celluloses. 
